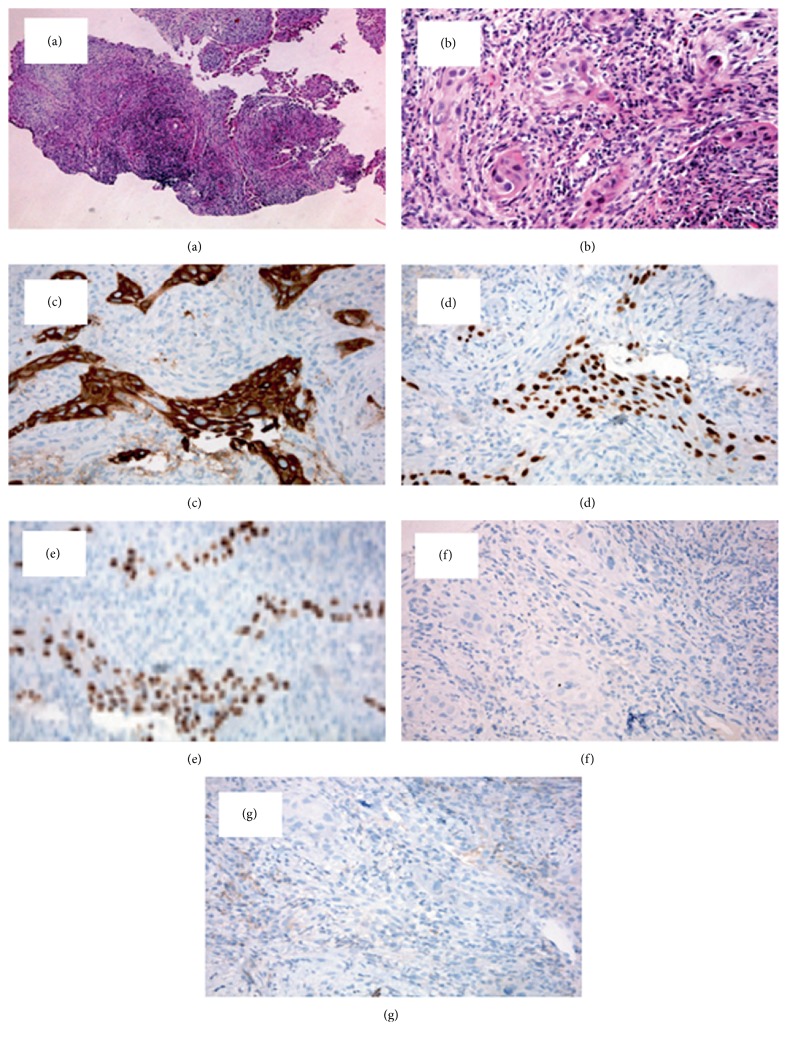# Lenvatinib Administered via Nasogastric Tube in Poorly Differentiated Thyroid Cancer

**DOI:** 10.1155/2019/6831237

**Published:** 2019-09-18

**Authors:** Eleonora Molinaro, David Viola, Nicola Viola, Pierpaolo Falcetta, Francesca Orsolini, Liborio Torregrossa, Paola Vagli, Alessandro Ribechini, Gabriele Materazzi, Paolo Vitti, Rossella Elisei

**Affiliations:** ^1^Unit of Endocrinology, Department of Clinical and Experimental Medicine, University Hospital of Pisa, Pisa 56124, Italy; ^2^Unit of Pathology, Department of Surgical, Medical, and Molecular Pathology and Critical Care Medicine, University Hospital of Pisa, Pisa 56124, Italy; ^3^Diagnostic and Interventional Radiology, University Hospital of Pisa, Pisa 56124, Italy; ^4^Endoscopic Section of Pneumology, University Hospital of Pisa, Pisa 56124, Italy; ^5^Unit of Surgery, Department of Surgical, Medical, and Molecular Pathology and Critical Care Medicine, University Hospital of Pisa, Pisa 56124, Italy

## Abstract

**Background:**

The tyrosine kinase inhibitors (TKIs) are indicated for the treatment of locally advanced or metastatic progressive thyroid carcinoma (CDT), refractory to radioactive iodine. The following report describes the efficacy of lenvatinib administered through a nose-gastric tube (SNG) in a patient affected with a poorly differentiated thyroid carcinoma (PDTC) which determined a stenosis of the esophagus.

**Material and Methods:**

A patient was followed up for papillary thyroid carcinoma follicular variant (T3NxMx), subjected to total thyroidectomy and treated with iodine-131 radio metabolic therapy. Two years after surgery, following the onset of dysphonia and dysphagia, patient was submitted to a computed tomography (CT) scan of the neck that showed the presence of a lesion of 6 × 2.5 × 3.5 cm, which determined trachea deviation and cervical esophagus compression. The biopsy indicated the presence of PDTC, triggering tracheal lumen reduction and sub-stenosis of the cervical esophagus for an ab-extrinsic compression. A nose-gastric tube (SNG) was placed and lenvatinib was started at a dose of 20 mg/day, administered via this probe after opening the capsules and diluting the drug in 10 ml of saline solution.

**Results:**

One month later, CT showed a significant cervical lesion reduction. Bronchoscopy confirmed tracheal infiltration, but the residual caliber was improved from 50% to 75%. At the esophagogastroduodenoscopy (EGDS), the sub stenosis of the cervical esophagus was no longer appreciated; however, a double perforation of the esophagus was found, without fistula.

**Conclusion:**

Lenvatinib therapy is effective also when administered via SNG. Our result is of particular relevance in the management of thyroid cancer patients, especially in the presence of subjects unable to swallow. Further studies are needed to validate the administration of lenvatinib by SNG, in order to extend the indications to this alternative administration way, beside the oral one.

## 1. Introduction

Poorly differentiated thyroid cancer (PDTC) is a rare aggressive endocrine neoplasm accounting for about 5% of all thyroid tumours. It was first described, independently, by Sakamoto et al. [1] and Carcangiu et al. [2], in 1983 and 1984, respectively. It shows morphological and behavioral features between those of well differentiated thyroid cancer (WDTC) and anaplastic thyroid cancer (ATC) and it may arise, de novo, in a thyroid gland, or from preexisting well-differentiated follicular and papillary carcinomas. Several studies showed a poorer prognosis for patients with PDTC compared to those with DTC, with a 10-year recurrence rate near 30% [3, 4]. Extra-thyroidal extension has negative impact on prognosis, with 10-year overall survival rates of 45% [5]. The incidence of extra-thyroidal extension varies, with a range from 6% to 13%. Trachea and esophagus were involved in 37% and 21% of patients with invasive thyroid cancer, respectively, as reported by McCaffrey et al. [6]

Surgery remains the mainstay for locally advanced thyroid cancer. However, the resection of vital neck structures can be associated with significant morbidity. For patients with advanced PDTC who are not suitable for surgical resection or radioiodine therapy, there are only few therapeutic strategies available. These patients could benefit of the new treatments, like the tyrosine-kinase inhibitors (TKI) [7, 8].

Lenvatinib, an oral multi-kinase inhibitor, has shown improvement in the progression-free survival and response rate among patients with iodine-131 (131-I) refractory (RAI refractory) thyroid cancer, including PDTC, compared to those receiving placebo [9]. Moreover, the drug was also able to reduce the tumoral lesions size as demonstrated by the significantly better objective response rate (ORR) in respect to patients treated with placebo [9].

We report a case of a PDTC showing an extremely aggressive behavior with tracheal and esophageal invasion, treated lenvatinib mesilate (Lenvima, Eisai Inc.), diluted in saline solution and administered via nasogastric feeding tube. The interest of this case report is that, although the company does not provide any indication for this administration route, the drug was effective in reducing the tumoral mass.

## 2. Patient

In December 2015, a 62-year-old female patient, underwent to a total thyroidectomy, following the finding of malignant cytology (TIR5) on thyroid nodule of the right lobe, and most likely malignant cytology (TIR4) on the nodule of the left lobe. Histological examination showed a papillary thyroid carcinoma (1.7 cm), multifocal, follicular variant with focal oxyphilous differentiation with involvement of the posterior peri-thyroid adipose tissue (pT3NxMx). In March 2016, 80 mCi of 131-I was administered for ablative purpose, with a whole body scan (WBS) documenting anterior cervical uptake; serum thyroglobulin was 0.25 ng/ml with AbTg 169 UI/ml. In December 2017, two years after the intervention, the patient came to our attention for the development of dysphagia and dysphonia, and underwent to an otorhinolaryngoiatric examination that documented the vocal cord paralysis, in the para-median position. Bronchoscopy also showed circumferential infiltration of the trachea, with 50% lumen reduction.

**Figure 1:**
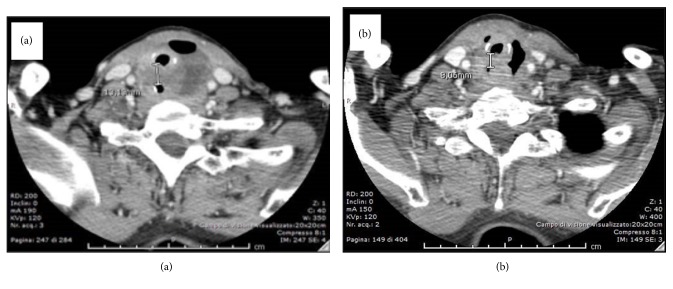


The esophagogastroduodenoscopy (EGDS) showed the sub-stenosis of the cervical esophagus due to an ab-extrinsic compression. A 4.9 mm nose-gastric tube (SNG) placement was performed and set, by endoscopic guidance. The neck CT documented a local recurrence of the disease, at the lower portion of the left thyroid loggia, which appeared as a fluid-air collection with necrotic evolution of 6 × 2.5 × 3.5 cm and which determined deviation and compression of the trachea and the proximal portion of the esophagus and showing no cleavage planes from cricoid cartilage, first tracheal rings and esophagus (Figure 1(a)). A biopsy was performed and histological examination documented morphological findings consistent with PDTC with squamous cells (Figure 2(a)–2(g)). The immunohistochemistry was positive for CK7, p40, and p63, focally positive for PAX-8 and negative for Tg and TTF-1. The proliferation index Ki-67 (MIB-1) was 30% and V600E mutation of BRAF was found. Radiotherapy was excluded due to the necrotic appearance of the lesion.

Lenvatinib therapy was immediately started and administered at a dose of 20 mg/day, via SNG, to our inpatient, opening the drug capsule and diluting the inside powder in 10 ml of saline solution; no special washes were made after the drug was administered. After one month from the start of therapy, the patient was re-evaluated. Neck CT showed the reduction of the fluid component, volume, and compressive effects of the cervical necrotic pathology (Figure 1(b)).

The bronchoscopy confirmed the paralysis of the left vocal cord, but the residual caliber in the most stenotic tracheal tract was increased from 50% up to 75%. The EGDS no longer documented the sub stenosis of the cervical esophagus, which was now traversed with the 6.5 mm instrument. However, a double esophageal perforation was reported at about 21 cm from the nostrils (without fistulous tract with the trachea).

In light of this finding, the drug was discontinued for a week, and the following control with EGDS showed the presence of a single perforation. Lenvatinib was restarted at a lower dose of 10 mg/day and the SNG was kept in place to continue the enteral nutrition. After one month, the second perforation appeared to be reduced but still present. Unfortunately, the patient died one month later for pulmonary embolism.

## 3. Discussion

Lenvatinib is an oral, multi-target, inhibitor of vascular endothelial growth factor receptors (VEGFR) 1, 2, and 3, fibroblast growth factor receptors 1–4, platelet-derived growth factor receptor alpha, and of the RET and KIT signaling pathways [10]. Its anti-tumoral activity in RAI-refractory thyroid cancer has been clearly demonstrated in the SELECT phase III clinical trial [9] and the drug has been approved for the treatment of these patients. Since our patient was unable to swallow, it was decided to administer the drug, lenvatinib, after capsules opening, via SNG. The treatment efficacy was confirmed by the important cyto-reduction observed at CT scan, performed after 1 month of therapy and by the reduction of tracheal and esophageal involvement observed at bronchoscopy and EGDS, respectively.

Common adverse events (AEs) related to lenvatinib treatment include hypertension, diarrhea, fatigue, rash, and palmar-plantar erythrodysestesia syndrome. Because of its strong anti-VEGFR activity, if the involvement of vital neck structures like trachea and esophagus by the tumor mass is of severe entity, the extensive necrosis caused by anti-angiogenic tyrosine kinase inhibitor therapy could lead to potentially, life threatening, AEs, like tracheoesophageal fistula and esophageal perforation [11]. Although the EGDS showed a compression of the esophagus but not an infiltration, our patient, responding to the therapy despite the different way of drug administration, developed the esophageal perforation, likely due to a microscopic infiltration. This is, unfortunately, a morbid condition with a mortality rate of 40–60% when the treatment is delayed [12], thus, despite the evidence of a significant reduction of the stenosis, we did not remove the SNG to avoid mediastinitis and, at the same time, we reduced the lenvatinib daily dose with the aim to let the perforations to heal. This strategy was demonstrated to be effective since, at the following EGDS control, one of the two perforations was healed and the other one reduced.

In our experience, this is the first reported case of PDTC, presenting to our observation after 2 years from surgical intervention and 131-I radio metabolic therapy, for which treatments were not applicable because of the disease aggressiveness and vital structures involvement. The multikinase inhibitor lenvatinib was extremely effective, even utilizing a not established administration method at the same dose that would have been chosen in the case of oral administration; we therefore do not recommend dose adjustments based on the type of route of administration. This case report has a pivotal role to encourage further specific studies in a wider population able to demonstrate the helpfulness of an alternative way of administration other than the oral one.

## Figures and Tables

**Figure 2:**